# Supplementary material for: Time-series transcriptome analysis identified differentially expressed genes in broiler chicken infected with mixed Eimeria species
Source: Front Genet. 2022 Aug 8;13:886781. doi: 10.3389/fgene.2022.886781 (PMC9393255; doi:10.3389/fgene.2022.886781)
Supplement: Supplementary file 2 [file DataSheet1.ZIP › 7dpi_KEGG.Gsea.1625115961950/KEGG_PROTEASOME.html]

Details for gene set KEGG\_PROTEASOME[GSEA]

|  || Dataset | TMM\_7dpi\_gct\_format\_7dpi\_gct\_format.Class\_4dpi.cls #PC\_versus\_NC.Class\_4dpi.cls #PC\_versus\_NC\_repos |
| Phenotype | Class\_4dpi.cls#PC\_versus\_NC\_repos |
| Upregulated in class | 1 |
| GeneSet | KEGG\_PROTEASOME |
| Enrichment Score (ES) | 0.53282297 |
| Normalized Enrichment Score (NES) | 2.1035953 |
| Nominal p-value | 0.0 |
| FDR q-value | 0.0017110616 |
| FWER p-Value | 0.0102 |
Table: GSEA Results Summary

  

Fig 1: Enrichment plot: KEGG\_PROTEASOME      
 Profile of the Running ES Score & Positions of GeneSet Members on the Rank Ordered List

  

| SYMBOL | TITLE | RANK IN GENE LIST | RANK METRIC SCORE | RUNNING ES | CORE ENRICHMENT || 1 | PSMB4 | na | 797 | 0.592 | 0.0325 | Yes |
| 2 | PSMB3 | na | 1011 | 0.520 | 0.1018 | Yes |
| 3 | PSMD14 | na | 1458 | 0.392 | 0.1302 | Yes |
| 4 | PSMD7 | na | 1583 | 0.359 | 0.1800 | Yes |
| 5 | PSMA3 | na | 1644 | 0.347 | 0.2332 | Yes |
| 6 | PSMD4 | na | 1678 | 0.337 | 0.2869 | Yes |
| 7 | PSMC2 | na | 2129 | 0.248 | 0.2908 | Yes |
| 8 | PSMB2 | na | 2130 | 0.248 | 0.3323 | Yes |
| 9 | PSMF1 | na | 2216 | 0.234 | 0.3644 | Yes |
| 10 | PSMD11 | na | 2315 | 0.219 | 0.3930 | Yes |
| 11 | PSMD12 | na | 2428 | 0.202 | 0.4174 | Yes |
| 12 | PSMA2 | na | 2432 | 0.202 | 0.4509 | Yes |
| 13 | PSMC5 | na | 2619 | 0.175 | 0.4646 | Yes |
| 14 | PSMB1 | na | 2867 | 0.143 | 0.4680 | Yes |
| 15 | PSMB7 | na | 2964 | 0.131 | 0.4820 | Yes |
| 16 | PSMA4 | na | 3006 | 0.126 | 0.4996 | Yes |
| 17 | PSMA5 | na | 3133 | 0.109 | 0.5074 | Yes |
| 18 | PSMD6 | na | 3303 | 0.086 | 0.5077 | Yes |
| 19 | PSMA6 | na | 3378 | 0.077 | 0.5144 | Yes |
| 20 | PSMD3 | na | 3477 | 0.065 | 0.5171 | Yes |
| 21 | POMP | na | 3524 | 0.061 | 0.5235 | Yes |
| 22 | PSMA1 | na | 3533 | 0.060 | 0.5328 | Yes |
| 23 | PSMC3 | na | 3758 | 0.034 | 0.5198 | No |
| 24 | PSMD2 | na | 3896 | 0.016 | 0.5111 | No |
| 25 | PSMC1 | na | 3990 | 0.006 | 0.5043 | No |
| 26 | PSMD13 | na | 3995 | 0.005 | 0.5049 | No |
| 27 | PSMA7 | na | 4170 | -0.014 | 0.4926 | No |
| 28 | PSME3 | na | 4197 | -0.017 | 0.4932 | No |
| 29 | PSMC6 | na | 4728 | -0.074 | 0.4613 | No |
| 30 | PSMD1 | na | 5925 | -0.194 | 0.3939 | No |
| 31 | PSME4 | na | 9838 | -0.680 | 0.1813 | No |
Table: GSEA details [plain text format]

  

Fig 2: KEGG\_PROTEASOME      
 Blue-Pink O' Gram in the Space of the Analyzed GeneSet

  

Fig 3: KEGG\_PROTEASOME: Random ES distribution      
 Gene set null distribution of ES for **KEGG\_PROTEASOME**
